# Genome-wide identification of short 2′,3′-cyclic phosphate-containing RNAs and their regulation in aging

**DOI:** 10.1371/journal.pgen.1008469

**Published:** 2019-11-13

**Authors:** Megumi Shigematsu, Keisuke Morichika, Takuya Kawamura, Shozo Honda, Yohei Kirino

**Affiliations:** Computational Medicine Center, Sidney Kimmel Medical College, Thomas Jefferson University, Philadelphia, Pennsylvania, United States of America; University of Pennsylvania School of Medicine, UNITED STATES

## Abstract

RNA molecules generated by ribonuclease cleavage sometimes harbor a 2′,3′-cyclic phosphate (cP) at their 3′-ends. Those cP-containing RNAs (cP-RNAs) form a hidden layer of transcriptome because standard RNA-seq cannot capture them as a result of cP’s prevention of an adapter ligation reaction. Here we provide genome-wide analyses of short cP-RNA transcriptome across multiple mouse tissues. Using cP-RNA-seq that can exclusively sequence cP-RNAs, we identified numerous novel cP-RNA species which are mainly derived from cytoplasmic tRNAs, mRNAs, and rRNAs. Determination of the processing sites of substrate RNAs for cP-RNA generation revealed highly-specific RNA cleavage events between cytidine and adenosine in cP-RNA biogenesis. cP-RNAs were not evenly derived from the overall region of substrate RNAs but rather from specific sites, implying that cP-RNAs are not from random degradation but are produced through a regulated biogenesis pathway. The identified cP-RNAs were abundantly accumulated in mouse tissues, and the expression levels of cP-RNAs showed age-dependent reduction. These analyses of cP-RNA transcriptome unravel a novel, abundant class of non-coding RNAs whose expression could have physiological roles.

## Author summary

With the advent and evolution of next-generation sequencing technology, efforts to identify and catalog the expressed RNA molecules have greatly advanced our understanding of RNA biology. However, the current standard RNA-seq methods, particularly those targeting short ncRNAs, do not fully capture all of the RNAs expressed but allow for some “escapers” to slip. RNAs generated by ribonuclease cleavage sometimes harbor a 2′,3′-cyclic phosphate (cP) at their 3′-ends, and those cP-containing RNAs (cP-RNAs) are one such escaper that are not ligated to a 3′-adapter and thus uncaptured by standard RNA-seq. Although an increasing number of studies has been suggesting their functional significances, cP-RNAs remained a hidden component in the transcriptome, infrequently recognized and characterized. In this study, we provide the first genome-wide analyses of short cP-RNA transcriptome across multiple mouse tissues. By using cP-RNA-seq technique that can specifically sequence cP-RNAs, we identified numerous novel cP-RNA species which are mainly derived from tRNAs, mRNAs, and rRNAs. cP-RNAs are generated by previously-uncharacterized, highly-specific RNA cleavage events between cytidine and adenosine, which is regulated through aging. These analyses of cP-RNA transcriptome unravel a novel, abundant class of non-coding RNAs whose expression could have physiological roles.

## Introduction

Cells express immensely diverse species of RNA molecules that play essential roles in numerous biological processes. With the advent of next-generation sequencing (NGS) technology, efforts to identify the expressed RNA molecules have greatly advanced our understanding of RNA biology [1]. Although NGS-based RNA-sequencing (RNA-seq) has become a ubiquitous tool in biological and medical research [2,3], the current standard RNA-seq methods, particularly those targeting short non-coding RNAs (ncRNAs), do not fully capture all of the RNAs expressed but allow for some “escapers” to slip by undetected. The RNAs containing a 2′,3′-cyclic phosphate (cP) at their 3′-end are one such escaper that are not ligated to a 3′-adapter during cDNA amplification procedure and thus uncaptured by standard RNA-seq. A cP end of RNA molecules, consisting of the phosphate linkage between the 2′- and 3′-positions of ribose, is mainly produced from RNA cleavage catalyzed by many endoribonucleases [4]. cP-containing RNAs (cP-RNAs) are not just accumulated as non-functional degradation products but have physiological roles in various biological processes. For example, Angiogenin (ANG), an RNase A-superfamily endoribonuclease, cleaves the anticodon-loop of tRNAs upon stress stimuli to produce functional tRNA halves known as tRNA-derived stress-induced RNAs (tiRNAs) [5]. The 5′-tiRNAs, corresponding to the 5′-tRNA halves, contain a cP at their 3′-end and promote the formation of stress granules, regulate translation, and trigger the cellular stress responses and apoptosis in neurodevelopmental disorders [6,7,8,9]. In breast and prostate cancer cells, sex hormone signaling pathways promote ANG-catalyzed tRNA cleavages, generating a distinct class of tRNA halves, the sex hormone-dependent tRNA derived RNAs (SHOT-RNAs) [10]. cP-containing 5′-SHOT-RNAs (5′-tRNA halves) have a functional significance in cell proliferation [10,11].

Given that cP-RNAs are expressed as functional molecules, capturing the entire repertoire of cP-RNAs would broaden the catalog of functional ncRNAs and reveal significant biological events that have been eluding standard RNA-seq. We recently developed “cP-RNA-seq,” which is able to specifically sequence cP-RNAs [10,12], and applied the method to a short RNA fraction of BT-474 breast cancer cells to identify 5′-SHOT-RNAs [10]. Because the previous work narrowly focused on 5′-SHOT-RNAs in only one cell line, cP-RNAs still largely remained a hidden component in the transcriptome. In this report, we expanded populations of RNA subjects and identified a comprehensive repertoire of short cP-RNAs in four different mouse tissues. A genome-wide in-depth view of cP-RNA transcriptome revealed that not only tRNAs but also other RNA species are widely, but specifically, cleaved between cytidine and adenosine for constitutive expression of diverse species of cP-RNAs. We further discovered age-dependent changes in the expression levels of cP-RNAs, implying the involvement of cP-RNA production in the aging process.

## Results and discussion

### cP-containing tRNA halves are abundantly and constitutively expressed in mouse tissues

**Fig 1:**
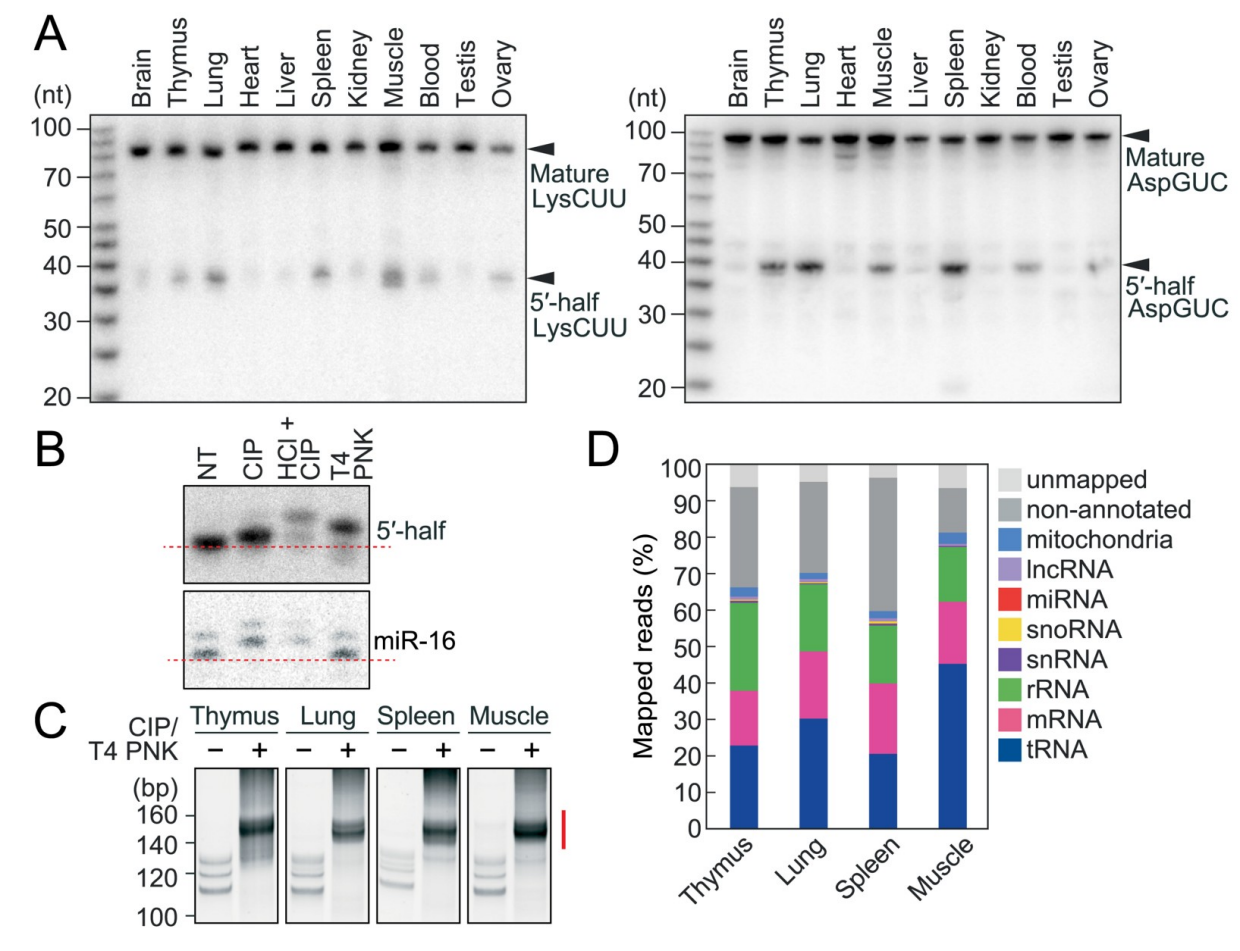
Sequencing of cP-RNAs in mouse tissues. **(A)** Total RNAs extracted from mouse tissues were subjected to northern blots for the 5′-halves of cyto tRNA^LysCUU^ and tRNA^AspGUC^. **(B)** Terminal structures of the 5′-tRNA half were analyzed enzymatically. Total RNA from the mouse lung was treated with CIP, T4 PNK, or acid followed by CIP treatment (HCl + CIP). NT designates the non-treated sample used as a negative control. The treated total RNA was subjected to northern blots targeting the 5′-tRNA^AspGUC^ half and microRNA-16 (miR-16). miR-16 was investigated as a control RNA containing 5′-P and 3′-OH ends. **(C)** Gel-purified 20–45-nt RNAs were subjected to cP-RNA-seq, which amplified 140–160-bp cDNA products (5′-adapter, 55 bp; 3′-adapter, 63 bp; and thereby estimated inserted sequences, 22–42 bp). The cDNAs in the region designated with a red line were purified and subjected to Illumina sequencing. **(D)** Proportion of cP-RNAs annotated to the indicated RNAs.

Because ANG-generated 5′-tRNA halves contain a cP, exploration of the accumulation of cP-RNAs in mouse tissues began with analyses of tRNA halves. Northern blots for cytoplasmic (cyto) tRNA^LysCUU^ and tRNA^AspGUC^, both of which abundantly produce tRNA halves in human breast cancer cells [10], revealed constitutive expression of both the 5′- and 3′-halves in various mouse tissues (**Figs 1A and S1A**). Although the levels of mature tRNAs were comparable across all the examined tissues, tRNA halves showed differential expression patterns among the tissues, implying a regulated biogenesis of tRNA halves. The abundant accumulation of 5′-tRNA halves found in the spleen and less abundant accumulations in other tissues, such as the brain, heart, liver, and testis, are consistent with previous reports [13,14]. To confirm the presence of a cP in the 5′-tRNA halves, total RNA from mouse lungs was treated with T4 polynucleotide kinase (T4 PNK; removes 3′-P/cP), calf intestinal phosphatase (CIP; removes 5′-P/3′-P, but not a cP), or acid followed by CIP treatment (removes 5′-P/3′-P/cP), and then the mobility of the bands of the 5′-tRNA half was examined by northern blots, as previously described [10,12]. As shown in **Fig 1B**, the band of the 5′-tRNA half shifted up by CIP treatment, suggesting the presence of a P in the 5′-tRNA half. T4 PNK treatment also shifted the band up, and further up-shift was observed by the acid plus CIP treatments, indicating the additional presence of a cP in the 5′-tRNA half. These results validated that, as observed for the 5′-tRNA halves in human cell lines [10], the 5′-tRNA halves in mouse tissues contain a P at their 5′-end and a cP at their 3′-end.

### Selective amplification and sequencing of short cP-RNAs expressed in mouse tissues

To capture an entire picture of short cP-RNAs, we selected four tissues (thymus, lung, spleen, and skeletal muscle) that exhibited an abundant expression of 5′-tRNA halves (**Fig 1A**). The four tissues commonly showed accumulations of short RNAs that were expected to include 5′-tRNA halves (**S1B Fig**), whereas we did not observe such short RNA accumulation in HEK293T and HeLa cells (**S1B Fig**), implying that the constitutive accumulation of short RNAs might be tissue specific. We chose to focus on the 20 to 45-nucleotide (nt) RNAs which were gel-purified and subjected to cP-RNA-seq [10,12]. The method successfully amplified ~140 to 160-bp cDNA products (inserted sequences without adapters were estimated to be 22–42 bp) whose dependency on T4 PNK treatment indicates that, as expected, the amplified bands were derived from cP-RNAs (**Fig 1C**). Illumina sequencing of the amplified bands from the respective four tissues yielded approximately 38–47 million raw reads, of which >90%–97% were extracted as the cP-RNA reads with a length of 20–45 nt (**S1 Table**). Read lengths of the extracted cP-RNAs similarly showed broad distribution patterns in different tissues (**S2 Fig**). Mapping the cP-RNA reads to the mouse ncRNAs and genome unexpectedly revealed that not only tRNAs but also the other RNA species serve as abundant substrates for cP-RNA production (**Fig 1D**). Although tRNA-derived reads were the most abundant cP-RNA species and occupied a substantial proportion of cP-RNAs, ~55%–80% reads are mapped to other regions of the genome, of which messenger RNAs (mRNAs) and ribosomal RNAs (rRNAs) were particularly rich sources of cP-RNAs (**Fig 1D and S1 Table**).

### tRNA-derived cP-RNAs are generated from focal cleavage of anticodon-loop and 3′-terminal CCA sequence of tRNAs

**Fig 2:**
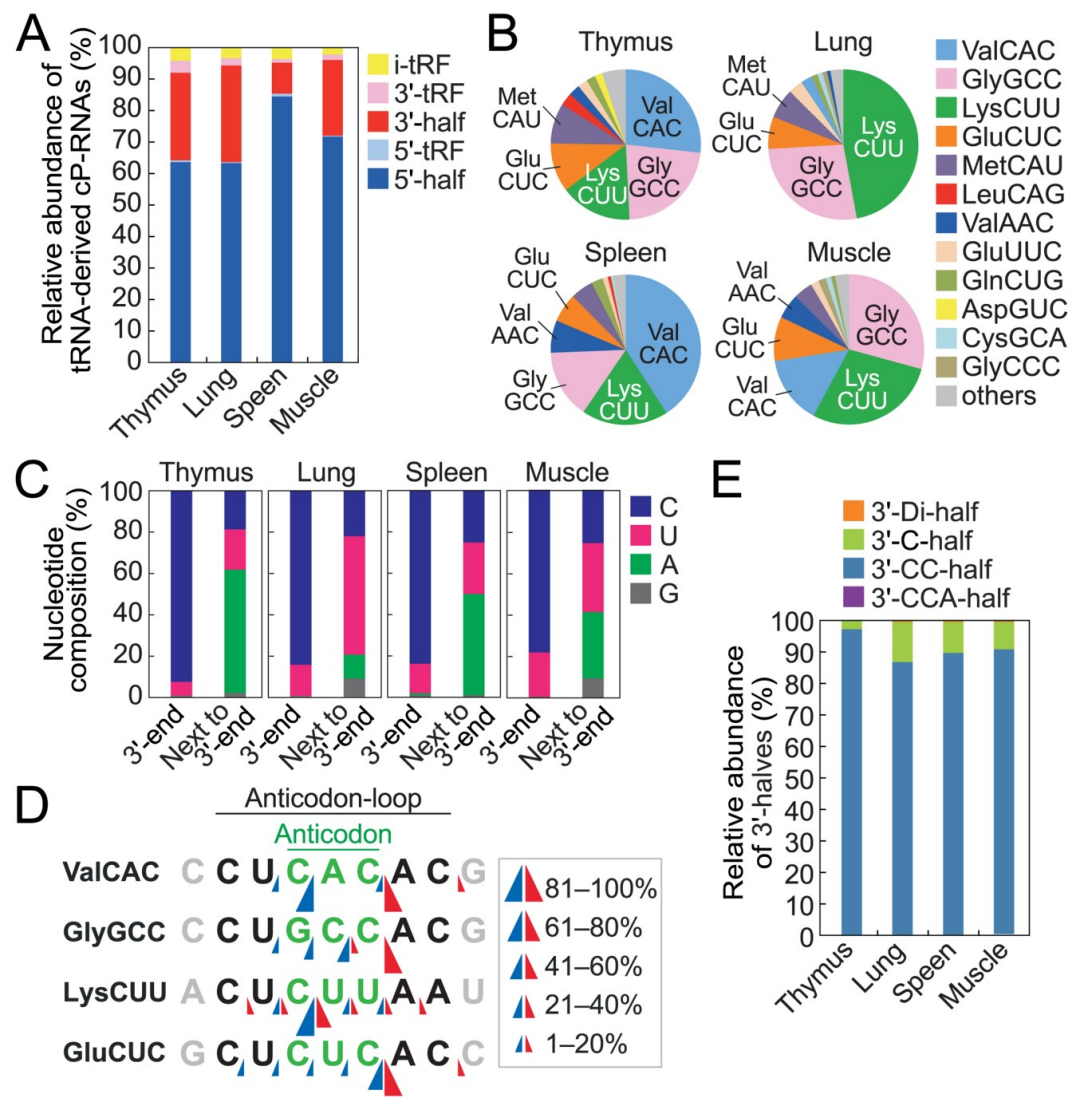
Analyses of tRNA-derived cP-RNAs. **(A)** Proportion of tRNA-derived cP-RNAs classified into the indicated subgroups of tRNA-derived ncRNAs shown in **S3A Fig**. **(B)** Proportion of the 5′-half-reads derived from respective cyto tRNA species. **(C)** Compositions of the 3′-terminal nucleotide of the 5′-halves and its 3′-adjacent nucleotide (potential 5′-terminal nucleotide of the 5′-cleavage product) in the anticodon-loop of tRNAs. **(D)** Cleavage sites in the anticodon-loops in the thymus were predicted based on the 3′-terminal positions of the 5′-halves (blue arrow heads) and the 5′-terminal positions of the 3′-halves (red arrow heads). The results for the other three tissues are shown in **S3B Fig**. **(E)** Proportion of the 3′-halves classified into the indicated subgroups shown in **S3A Fig**. The numerical data is shown in **S2 Table**.

tRNA-derived ncRNAs can be classified into 5′-tRNA half, 3′-tRNA half, 5′-tRF, 3′-tRF, and internal-tRF (i-tRF) [15,16,17,18] (**S3A Fig**). tRNA-derived cP-RNAs were mostly 30–40 nt long (**S2 Fig**) and the majority were 5′-halves (**Fig 2A**) as expected. Considering that the mouse genome encodes 55 cyto tRNA isoacceptors with different anticodon sequences [19], the identified 5′-halves were derived from a rather focused subset of tRNAs, such as cyto tRNA^ValCAC^, tRNA^GlyGCC^, tRNA^LysCUU^, and tRNA^GluCUC^, which are in aggregates the sources of 75%–84% of the identified 5′-halves (**Fig 2B**). The 3′-terminal nucleotides of the 5′-halves had a strongly biased composition in being pyrimidines (C > U), which was a stable property in all four examined tissues (**Fig 2C**). The anticodon-loop nucleotides that are one position downstream of the 3′-end showed biases of A and U, allowing us to predict that the anticodon-loop cleavage mainly occurs between C and A/U to produce cP-containing 5′-halves (**Figs 2C and 2D and S3B**).

Among all species of tRNAs, tRNA^HisGUG^ is unique in that it contains an additional nucleotide at nucleotide position (np; according to the nucleotide numbering system of tRNAs [20]) –1 of its 5′-end. Our recent TaqMan RT-qPCR analyses revealed that, in BT-474 cells, the majority (~60%) of cyto tRNA^HisGUG^ contains G_–1_, but a significant proportion contains U_–1_ or lacks the –1 nucleotide, resulting in G_1_ as a 5′-terminal nucleotide [21]. As shown in **S3C Fig**, a majority of the identified 5′-halves of cyto tRNA^HisGUG^ contained G_–1_, but the 5′-halves containing U_–1_ or lacking –1 nucleotide (G_1_) were also substantially detected, suggesting that the presence of U_–1_ as well as G_–1_ is a conserved feature in mouse cyto tRNA^HisGUG^, which is retained in the identified 5′-halves.

It was unexpected that a substantial proportion (9%–31%) of tRNA-derived cP-RNAs were occupied by 3′-halves. All three tRFs (5′-, 3′-, and i-tRFs) were identified as minor cP-containing species compared to the tRNA halves. When subclassified into the four subgroups (3′-CCA-/3′-CC-/3′-C-/3′-Di-halves; **S3A Fig**), nearly all (89%–98%) of the identified 3′-halves were 3′-CC-halves (**Fig 2E and S2 Table**), suggesting that the 3′-terminal cP formation resulted from the cleavage between C_75_ and A_76_ of tRNAs. The *in vitro* ANG-catalyzed cleavage between C_75_ and A_76_ was previously reported [22], and our results might suggest the occurrence of cleavage *in vivo*. The 3′-CC- and 3′-C-halves were identified as cP-RNAs also in human brain [23], suggesting the consistency between mouse and human tissues. The anticodon-loop cleavage sites predicted by the 3′-terminal position of the 5′-halves and the 5′-terminal position of the 3′-halves were sometimes consistent (**Figs 2D and S3B**). Therefore, specific tRNAs are likely to be selected for cP-RNA production and at least some of their 5′- and 3′-half pairs could be concurrently produced by endonucleolytic cleavage at the anticodon-loops.

### mRNA-derived cP-RNAs are produced from a specific processing between adenine and cytosine

**Fig 3:**
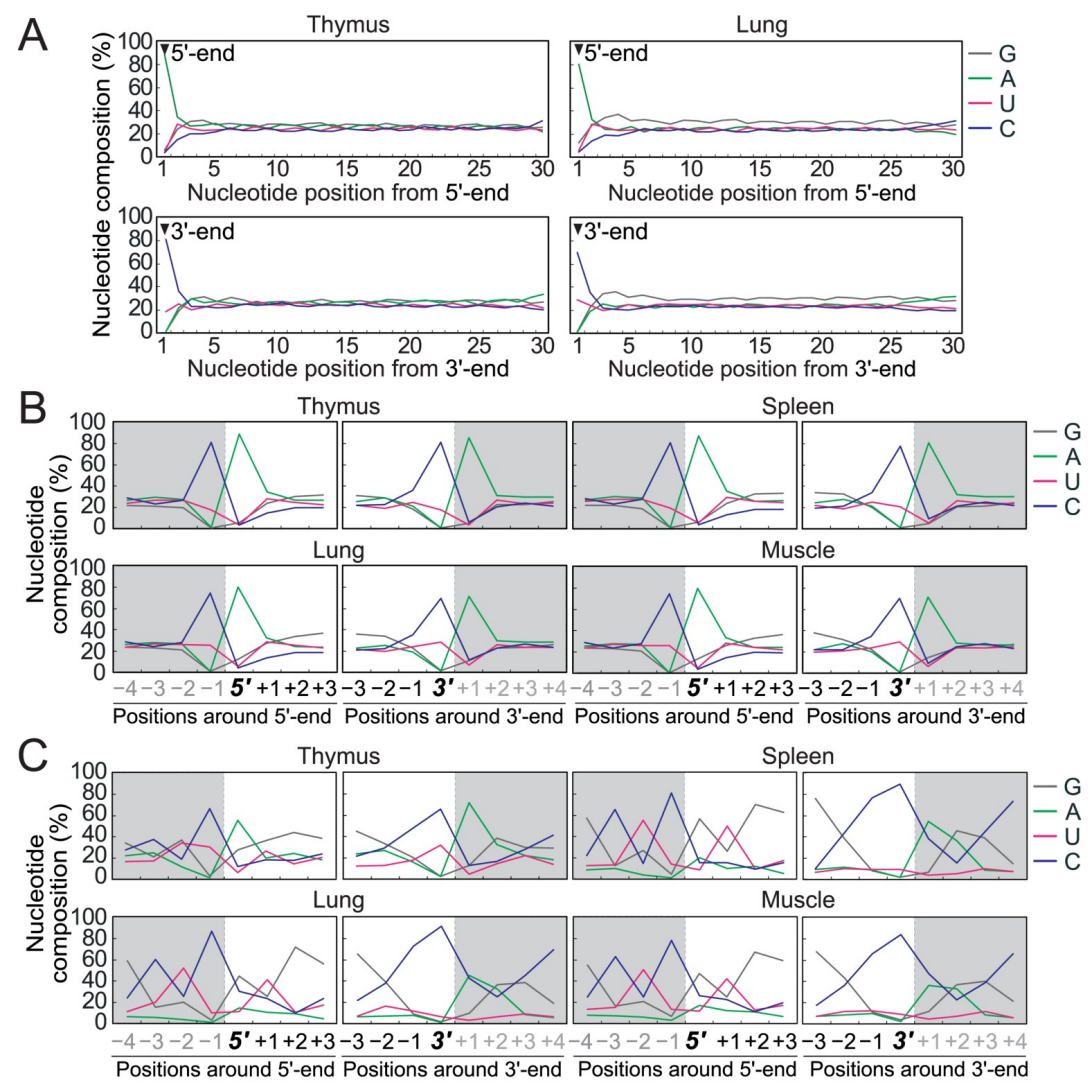
Nucleotide composition analyses of mRNA- and rRNA-derived cP-RNAs. **(A)** Nucleotide compositions of the first 30 nucleotides from the 5′-end (upper) or 3′-end (lower) of the mRNA-derived cP-RNAs in the thymus and lung. The results for the other two tissues are shown in **S4B Fig**. **(B)** Nucleotide compositions around the 5′- and 3′-ends of mRNA-derived cP-RNAs. A dashed line separates upstream (-) and downstream (+) positions for the 5′-and 3′-ends, representing the cleavage site that generates mRNA-derived cP-RNAs (the regions outside of cP-RNA-generating regions are colored in grey). **(C)** Nucleotide compositions around the 5′- and 3′-ends of rRNA-derived cP-RNAs.

Although not anticipated, not only tRNAs but also other RNA species were identified as rich sources of cP-RNAs. mRNA-derived cP-RNAs were enriched in cP-RNA libraries from all four examined tissues, accounting for 15%–20% of all cP-RNA species (**Fig 1D**). The lengths of the mRNA-derived cP-RNAs were broadly distributed between 20–45 nt (**S2 Fig**). Both coding and untranslated regions of mRNAs became substrates for cP-RNAs (**S4A Fig**). Astonishing nucleotide composition biases were observed at both the 5′- and 3′-ends of mRNA-derived cP-RNAs in all four tissues (**Figs 3A and S4B**). The 5′-ends were mostly A (78%–89%), whereas the 3′-ends were mostly pyrimidines, and C (70%–81%) was especially enriched compared to U (17%–29%). None of the biases were observed in the nucleotides from the second position to the position preceding the 3′-ends. The nucleotides that are one position upstream of the 5′-ends and one position downstream of the 3′-ends showed clear A and C biases (**Fig 3B**), strongly suggesting that the mRNA-derived cP-RNAs are produced by an endoribonuclease that specifically recognizes and cleaves mRNAs between C and A residues.

**Fig 4:**
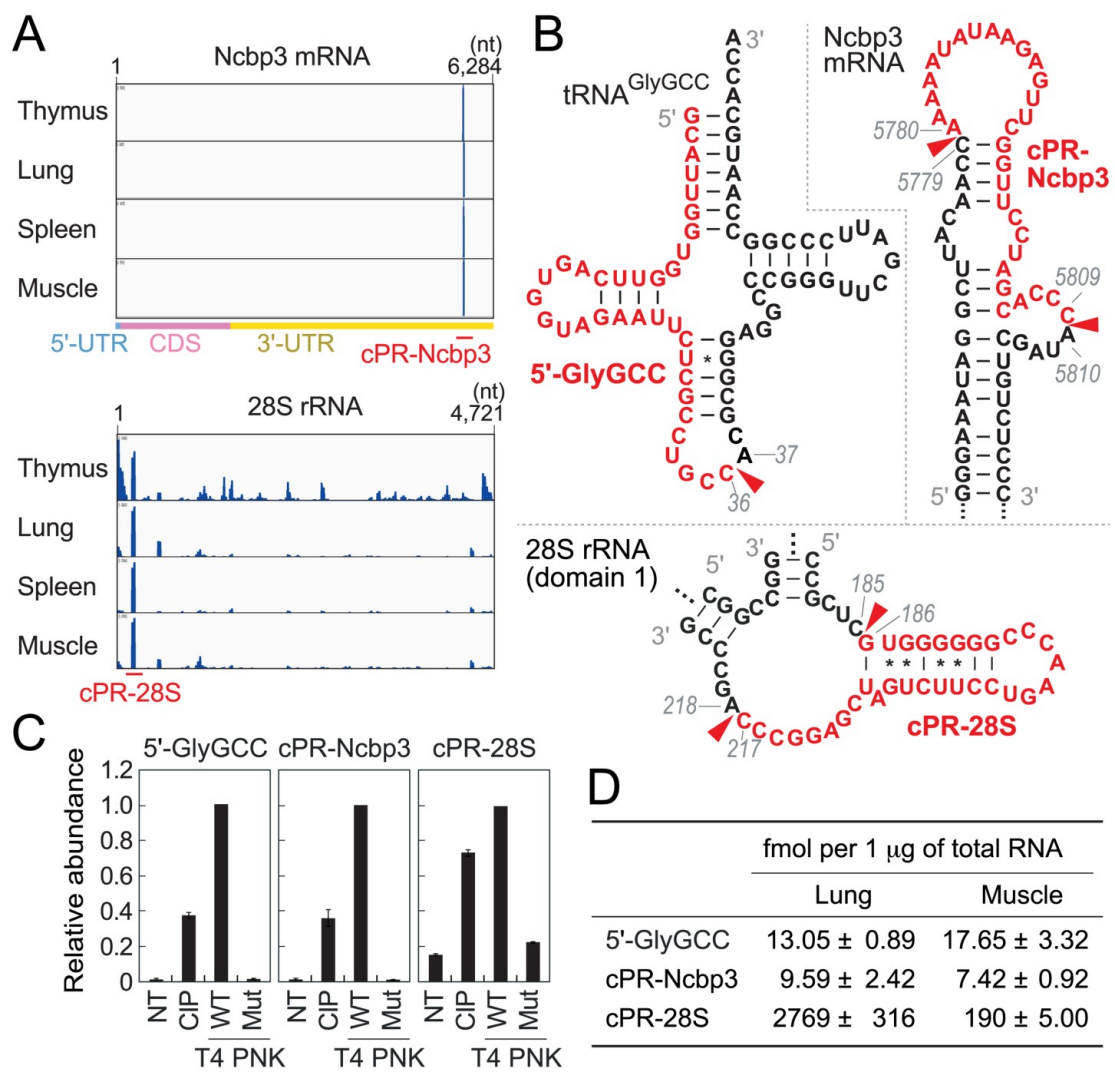
TaqMan RT-qPCR quantification of cP-RNAs. **(A)** The alignment patterns of cP-RNAs for Ncbp3 mRNA and 28S rRNA. The positions of representative cP-RNAs, cPR-Ncbp3 and cPR-28S, are indicated. **(B)** The regions from which 5′-tRNA^GlyGCC^ half (5′-GlyGCC), cPR-Ncbp3, and cPR-28S were derived are shown in red in the secondary structure of respective substrate RNAs. Secondary structure of Ncbp3 mRNA was predicted by ViennaRNA Package 2.0 [34]. **(C)** The total RNA from 24-week old mouse skeletal muscle, treated with CIP, wild-type (WT) T4 PNK, or mutant (Mut) T4 PNK lacking 3′-dephosphorylation activity, was subjected to TaqMan RT-qPCR. NT designates a non-treated sample used as a negative control. The amounts from WT T4 PNK-treated RNA were set as 1, and relative amounts are indicated. Averages of three experiments with SD values are shown. **(D)** The expression of cP-RNAs in the lung and skeletal muscle of 24-week old mice were quantified using TaqMan RT-qPCR. The cP-RNA amounts were estimated based on the standard curves shown in **S9 Fig**. Averages of three independent experiments with SD values are shown.

Major mRNA species producing cP-RNAs are not very consistent among different tissues (**S3 Table**), and the cP-RNAs were derived from many different regions throughout the mRNAs (**S4C Fig**). In the spleen, many abundant cP-RNAs were derived from hemoglobin mRNAs, which might be correlated to the role of the spleen in red blood cell turnover that requires the degradation of hemoglobin [24]. It should be noted that some mRNAs, such as Hist1h2br and B2m in the thymus (**S4C Fig**), showed very focal production of specific cP-RNAs, suggesting that the cP-RNA production and accumulation are not caused by random mRNA degradation. The most prominent example is Ncbp3 mRNA which commonly produces the most abundant mRNA-derived cP-RNA in all four tissues (**S3 Table**). Although there are 406 “CA” sequences (potential cleavage sites) throughout Ncbp3 mRNAs, almost all cP-RNAs are produced from only one site (position 5780–5809; the generated cP-RNA is termed cPR-Ncbp3) in the 3′-UTR (**Fig 4A**), where single-stranded loop regions are expected to be formed (**Fig 4B**). The common and specific production of cP-RNAs might suggest that these RNAs are produced as functional molecules.

### cP-RNAs produced from other RNA species are also produced from a specific processing between adenine and cytosine

rRNA-derived cP-RNAs, whose read lengths were broadly distributed between 20–45 nt (**S2 Fig**), were enriched at similar levels (15%–25% of all cP-RNA species; **Fig 1D**) with mRNA-derived cP-RNAs. All four rRNA species (28S, 18S, 5.8S, and 5S) produce cP-RNAs (**S5A Fig**), and the most abundant cP-RNA species were derived from 28S rRNA (**S4 Table**). Nucleotide biases observed in mRNA-derived cP-RNAs were retained mostly in rRNA-derived cP-RNAs; the 3′-end and a nucleotide upstream of the 5′-end were strongly biased to C and U (C > U) (**Fig 3C**). The 5′-end and a nucleotide downstream of the 3′-end were not only biased to A, but also to G, and sometimes to U. Alignment visualization showed production of specific cP-RNA species from rRNAs (**Figs 4A and S5B**), suggesting their controlled biogenesis mechanisms. In 28S rRNA, the positions 186–217 was the most abundant cP-RNA-producing region (**Fig 4A**), and the representative cP-RNA from the region, termed cPR-28S, is produced from the cleavages between C_185_ and G_186_, and C_217_ and A_218_ in domain 1 (**Fig 4B**). Cleavage positions reside in the loop regions of 28S rRNA, possibly because the regions are highly accessible to endoribonucleases.

cP-RNAs from small nuclear RNAs (snRNAs), small nucleolar RNAs (snoRNAs), microRNAs (miRNAs), and long ncRNAs (lncRNAs) showed terminal nucleotide bias to A and C for their 5′- and 3′-end, respectively, in all four tissues (**S6 and S7 Figs**). Moreover, not only the cP-RNAs derived from the nuclear genome but also the cP-RNAs derived from the mitochondrial genome showed similar nucleotide biases (**S8 Fig**). Although those cP-RNAs were not abundant, the consistent nucleotide biases suggest that cP-RNAs are produced from a wide variety of RNA species through identical RNA processing events mainly between cytidine and adenosine.

### The identified cP-RNAs are abundantly accumulated in mouse tissues

To biochemically validate that the identified sequences are expressed as cP-RNAs, we focused on the three representative cP-RNAs, 5′-half of tRNA^GlyGCC^ (5′-GlyGCC), cPR-Ncbp3, and cPR-28S (**Fig 4B**), which are one of the most abundantly sequenced species among tRNA-, mRNA-, and rRNA-derived cP-RNAs, respectively. To analyze 3′-terminal structures of the three species, total RNA from mouse skeletal muscle was treated with CIP or T4 PNK. A mutant T4 PNK, which lacks 3′-dephosphorylation activity [25], was also used as a control. Subsequently, efficiency of the ligations between each target RNA and a 3′-adapter was examined by TaqMan RT-qPCR as described in [26]. As shown in **Fig 4C**, all three target RNAs exhibited the highest amplification signals upon T4 PNK treatment, indicating that the major 3′-terminal form of the identified sequences are a cP. CIP treatment showed certain signal levels, suggesting co-expression of 3′-P-containing RNAs as a minor form or occurrence of hydrolysis reaction converting a cP to a P during experimental procedures.

Using their specific quantification by TaqMan RT-qPCR, we explored absolute amounts of the three cP-RNAs expressed in mouse lung and skeletal muscle. The calculation of the amounts was based on the standard curve from synthetic RNAs which showed excellent linearity between input amounts and amplification signals (**S9 Fig**). The determined abundances of the three cP-RNAs per 1 μg of total RNA were summarized in **Fig 4D**. Whereas the amounts of 5′-GlyGCC and cPR-Ncbp3 were comparable between lung and muscle, the amount of cPR-28S in lung is ~14-fold higher than that in muscle. Considering previous reports estimating that 1 μg of total RNA from mouse liver contains 0.005–8.8 fmol of miRNA [27], the accumulation of cP-RNAs in mouse tissues are estimated to be much more abundant than that of miRNAs.

### Age-dependent decrease in the expression levels of cP-RNAs

**Fig 5:**
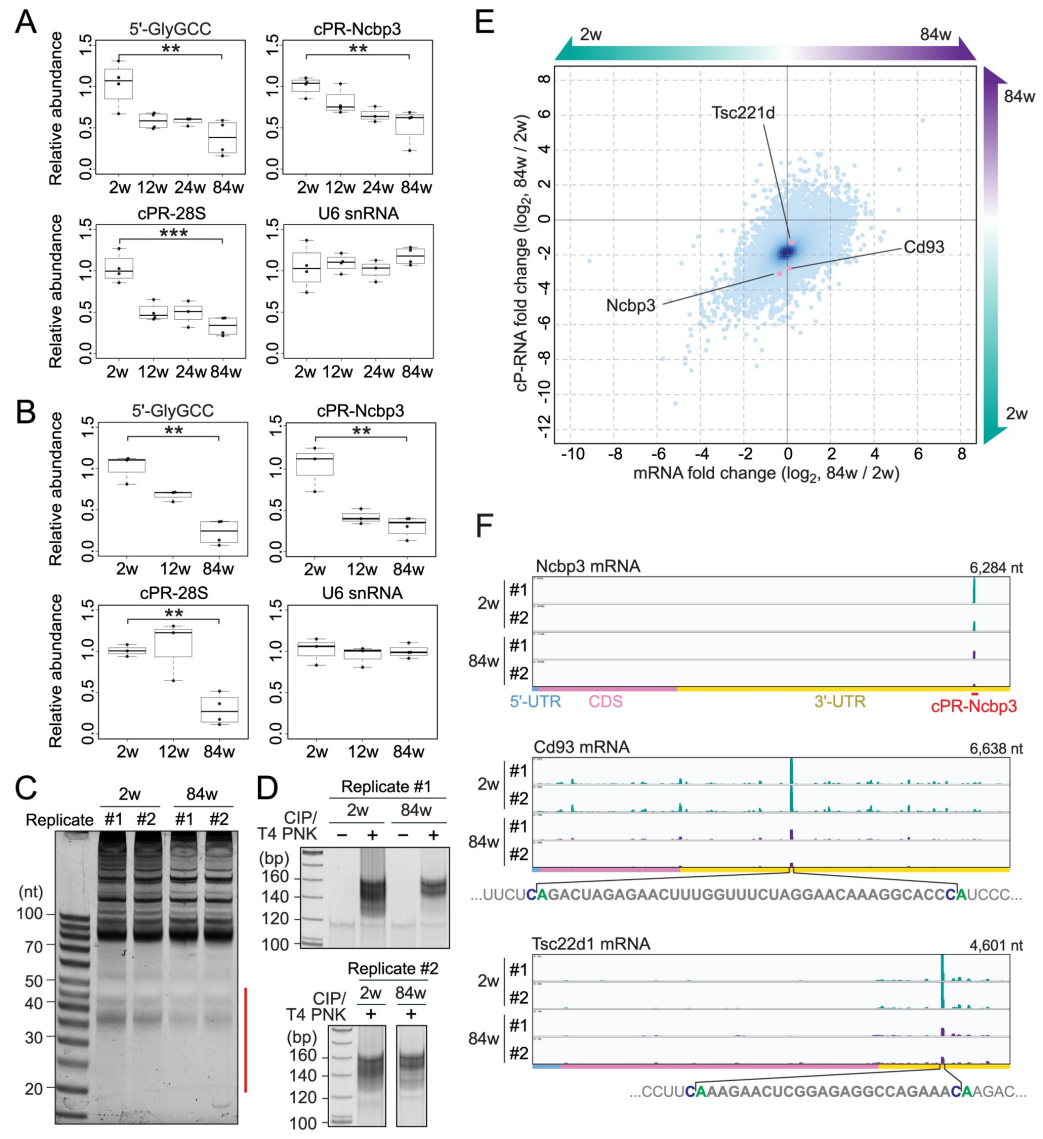
Expression of cP-RNAs in tissues from mice with different ages. **(A)** Total RNAs from skeletal muscle of 2–84-week old mice were subjected to TaqMan RT-qPCR for quantification of the indicated cP-RNAs. U6 snRNA was quantified as the control whose expression is unchanged through aging. **P < 0.01, ***P < 0.001 by *t*-test. **(B)** Total RNAs from lung of 2–84-week old mice were subjected to cP-RNA quantification. (**C**) Total RNAs from the biological duplicates of lung from 2- and 84-week old mice were subjected to denaturing PAGE and stained by SYBR Gold. The RNAs in the region designated with a red line were purified and subjected to cP-RNA-seq. (**D**) cDNAs amplified by cP-RNA-seq. **(E)** A scatter plot representing the fold changes of the read numbers for each mRNA in 84-week samples, with respect to those in 2-week samples, from cP-RNA-seq (y-axis) and mRNA-seq (x-axis). The plots of the three mRNAs, whose alignment patterns are shown in **Fig 5F**, are highlighted. **(F)** The alignment patterns of cP-RNAs for Ncbp3, Cd93, and Tsc22d1 mRNAs. The viewer height in each panel was adjusted based on the normalization by the reads of a spike-in cP-RNA.

To explore physiological relevance of cP-RNA expression, TaqMan RT-qPCR quantification of the three cP-RNAs were performed for skeletal muscle of mice with four different ages, 2, 12, 24, and 84 weeks. While a control U6 snRNA showed consistent expression levels in all the examined mice, the expression levels of all three cP-RNAs were reduced along with aging, and the differences between 2- and 84-week old mice were especially apparent (**Fig 5A**). The cP-RNA levels in 12- and 24-week old mice, both of which are considered as adult mice, did not show a significant change between them and were in-between those of 2- and 84-week old mice. To ask whether the phenomenon is conserved in different tissues, we further investigated lung of mice with different ages, showing the reduction of the expression levels of all three cP-RNAs through aging (**Fig 5B**). Total RNA staining pattern further suggested global reduction of the expression of 30–45-nt short ncRNAs, which are expected to contain cP-RNAs, through aging (**Fig 5C**).

To investigate potential relationship between cP-RNAs and their substrate RNAs in expressional regulation through aging, we focused on mRNA-derived cP-RNAs. Total RNAs from lung of 2- and 84-week old mice were subjected to cP-RNA-seq and mRNA-seq for identification of mRNA-derived cP-RNAs and their substrate mRNAs, respectively. Consistent with the downregulated expression of cP-RNAs in older mice (**Fig 5A–5C**), cP-RNA-seq amplified less abundant cDNAs from the 84-week old mice compared to the 2-week old mice (**Fig 5D**). Illumina sequencing of the cDNAs yielded ~23–29 million raw reads, of which ~3–6.5 million reads were extracted as the reads of mRNA-derived cP-RNAs (**S5 Table**). After normalization based on the read number information of a spike-in cP-RNA, fold changes of cP-RNA read numbers of 84 week samples, with respect to those of 2 week samples, were compared with fold changes of mRNA-seq read numbers for each mRNA in a scatter plot (**Fig 5E**). Strong aggregations of the spots appeared at the point where the levels of cP-RNAs were 4-fold reduced in 84-week samples compared to 2-week samples without changes in the levels of their substrate mRNAs. Consistent with the results of TaqMan RT-qPCR (**Fig 5A and 5B**), reduction of cPR-Ncbp3 was confirmed in the scatter plot (**Fig 5E**). As representative results, normalized alignment patterns of cP-RNAs in the three selected mRNAs, Ncbp3, Cd93, and Tsc22d1, are shown in **Fig 5F**. Specific productions of cP-RNAs through cleavage between C and A residues and their reduction in 84-week samples were observed. Together with the decreased C and A nucleotide biases in 84-week samples (**S10 Fig**), these results suggest that the expression of cP-RNAs is decreased through aging, which is independent of the levels of substrate RNAs but is caused by reduced C-A cleavage activity for cP-RNA productions or by reduced stability of cP-RNAs.

### Conclusion

In this study, we unraveled a previously hidden layer of transcriptome by comprehensively identifying short cP-RNA species expressed in mouse tissues. Each cP-RNA library, obtained by cP-RNA-seq, commonly comprised previously uninterrogated cP-RNA species that are expected to be generated by specific RNA cleavage between cytidine and adenosine (**S11 Fig**). The endoribonuclease generating the identified cP-RNAs is unknown but should possess highly specific recognition and cleavage activity for the 5′-CA-3′ sequence. ANG might be the candidate enzyme because ANG produces cP-RNAs [10,28] and shows the highest activity toward the 5′-CA-3′ among four examined dinucleotides in a previous study [29]. Among other cP-generating ribonucleases, GCN4 shows high cleavage activity between C and A [30]. Because cP-RNAs are not evenly produced from overall sequences of substrate RNAs but rather derived from specific sites, the RNA cleavage activity to generate cP-RNAs might be exquisitely regulated. Further investigations are required to clarify the enzyme and its activity for the generation of the cP-RNAs identified in this study. Considering the already-proven functional significance of tRNA-derived cP-RNAs [6,7,8,9,10], it is not surprising that the identified cP-RNAs themselves have functional significance. Their abundance and expression from only specific sites of substrate RNAs might also imply their expression as functional molecules. The reduction of cP-RNA generation through aging implied its physiological relevance. It will be intriguing to examine further the cellular factors that influence, or are influenced by, the expression of cP-RNAs and their underlying molecular mechanisms, which will define cP-RNAs as an abundant class of functional non-coding RNAs.

## Materials and methods

### Ethics statement

All mouse experiments were conducted in compliance with the standards and guidelines of the National Institutes of Health (NIH) and were approved by the Institutional Animal Care and Use Committee at Thomas Jefferson University. Mice were euthanized by CO_2_ inhalation followed by tissue dissections.

### RNA isolation and enzymatic treatment

For cP-RNA-seq, tissues of thymus, lung, spleen, and skeletal muscle from hindlimbs were dissected from male C57BL/6J (B6) mice at approximately 3-months ages. The dissected tissues were homogenized in TRIsure (Bioline) using a glass homogenizer on ice, after which total RNA was extracted according to the manufacturer’s instruction. For TaqMan RT-qPCR, frozen tissues were crushed using a frozen mortar and a pestle, followed by total RNA extraction by TRIsure. To investigate the terminal structures of the 5′-tRNA half, total RNA was treated with CIP (New England Biolabs), T4 PNK (New England Biolabs), or acid (incubation in 10 mM HCl at 4°C for 3 h) followed by CIP treatment as previously described [10,12].

### Northern blot

Northern blot was performed as previously described [31] with the following antisense probes: 5′-tRNA^LysCUU^ half, 5′- GTCTCATGCTCTACCGACTG-3′; 3′-tRNA^LysCUU^ half, 5′-GCTCGAACCCACGACCCTGAGA-3′; 5′-tRNA^AspGUC^ half, 5′-GGGATACTCACCACTATACTAACGAGGA-3′; 3′-tRNA^AspGUC^ half, 5′- GAATCGAACCCCGGTCTCC-3; and miR-16, 5′-GCCAATATTTACGTGCTGCTA-3′.

### cP-RNA-seq

For four tissue samples (thymus, lung, spleen, and skeletal muscle), 20 to 45-nt RNAs were gel-purified from total RNA and subjected to cP-RNA-seq procedure as previously described [10,12]. The amplified cDNAs were gel-purified and sequenced using the Illumina HiSeq 2500 system at the Next-Generation Sequencing Core of the University of Pennsylvania.

For comparison between 2- and 84-week old mice, lung 20–45-nt RNAs were gel-purified together with a synthetic spike-in cP-RNA (5′-CAGUGGUGGGCCAGAUGUAAACAUUAGAUUGUUCUUG-cP-3′, synthesized by ChemGenes). cDNAs amplified by cP-RNA-seq procedure were sequenced using Illumina NextSeq 500 at the MetaOmics Core of Sidney Kimmel Cancer Center in Thomas Jefferson University.

### Bioinformatics analyses

The cP-RNA-seq libraries contain ~22–47 million raw reads (**S1 and S5 Tables**) and are publically available at NCBI’s Sequence Read Archive (accession No. SRR8164560, SRR8164561, SRR8164562, SRR8164563, SRR9651811, SRR9651812, SRR9651813, and SRR9651814). Before mapping, we used the cutadapt tool (DOI: http://dx.doi.org/10.14806/ej.17.1.200) to remove the 3′-adapter sequence. After selecting 20–45-nt reads, we used Rbowtie (1.15.1) [32] for the sequential mappings with one mismatch allowed. The reads were first mapped to 471 mature cyto tRNAs obtained from GtRNAdb [19], and then to mature rRNAs, to mitochondrial genome (GenBank DQ106412 sequence plus 22 mitochondrial tRNA sequences), to mRNAs of RefSeq with NM-staring accession numbers, and to whole genome. All the sequences and annotations, except for the mitochondrial genome, were derived from the Mus musculus GRCm38 (mm10) genome assembly. Nucleotide compositions were analyzed by running FastQC (https://www.bioinformatics.babraham.ac.uk/projects/fastqc/). For the nucleotide composition analyses of the sequences which are upstream or downstream of cP-RNA producing regions on the genome, 4-nt upstream/downstream sequences were retrieved by referring fasta files and subjected to FastQC analyses. The Integrative Genomics Viewer (IGV) [33] was used for visualizing the aligned reads.

For comparison between 2- and 84-week samples, the extracted 20–45-nt cP-RNA reads were first mapped to the sequence of the spike-in cP-RNA, followed by the sequential mappings as described above. The read numbers of the spike-in cP-RNA were used to calculate the normalized RPKM of each cP-RNA-producing mRNA. mRNA-seq was performed by Novogene and RPKM of each mRNA was also obtained. These RPKM values were used for calculations of fold-changes of 84-week samples, with respect to 2-week samples, and the values derived from cP-RNA-seq and mRNA-seq were compared in the scatter plot. The experiments of cP-RNA-seq and mRNA-seq were performed using biological duplicates and triplicates, respectively.

### Quantification of cP-RNAs by TaqMan RT-qPCR

TaqMan RT-qPCR for specific quantification of cP-RNAs was performed as previously described [10]. Briefly, to remove a cP from cP-RNAs, total RNA was treated with T4 PNK, followed by ligation to a 3′-RNA adapter by T4 RNA ligase. Ligated RNA was then subjected to TaqMan RT-qPCR using the One Step PrimeScript RT-PCR Kit (TaKaRa), 200 nM of a TaqMan probe targeting the boundary of the target RNA and 3′-adapter, and specific forward and reverse primers. The quantified cP-RNA levels were normalized to 5S rRNA levels. The sequences of the TaqMan probes and primers are shown in **S6 Table**. For the quantification of absolute amounts of cP-RNAs, each synthetic RNA was mixed with 1 μg of *E*. *coli* total RNA and the mixtures were used as the substrates to obtain standard curves shown in **S9 Fig**. In **Fig 5A and 5B**, Student’s *t*-tests were used to evaluate the differences between 2- and 84-week samples.

## Supporting information

S1 Fig RNAs of mouse tissues. (EPS)

S2 Fig Read-length distributions of cP-RNA sequences obtained by cP-RNA-seq. (EPS)

S3 Fig Analyses of tRNA-derived cP-RNAs. (EPS)

S4 Fig Analyses of mRNA-derived cP-RNAs. (EPS)

S5 Fig Analyses of rRNA-derived cP-RNAs. (EPS)

S6 Fig 5′- and 3′-terminal nucleotide compositions of the cP-RNAs derived from snRNAs, snoRNAs, miRNAs, and lncRNAs. (EPS)

S7 Fig Nucleotide compositions of the first 30 nucleotides from the 5′-end or the 3′-end of the lncRNA-derived cP-RNAs. (EPS)

S8 Fig Analyses of mitochondrial cP-RNAs. (EPS)

S9 Fig Standard curves for the quantification of cP-RNAs. (EPS)

S10 Fig Nucleotide composition analyses of mRNA-derived cP-RNAs. (EPS)

S11 Fig Schematic representation of the generation of cP-RNAs and its age-dependent changes. (EPS)

S1 Table Read numbers of cP-RNA libraries obtained from cP-RNA-seq for four mouse tissues. (XLSX)

S2 Table Proportion of the 3′-halves (%). (XLSX)

S3 Table Top 50 reads of mRNA-derived cP-RNAs. (XLSX)

S4 Table Top 50 reads of rRNA-derived cP-RNAs. (XLSX)

S5 Table Read numbers of cP-RNA libraries obtained from cP-RNA-seq for lung of 2 and 84 week-old mice. (XLSX)

S6 Table Sequences of TaqMan probes and primers for RT-qPCR. (XLSX)
